# Evaluation of Neighborhood Socio-Economic Status, as Measured by the Delphi Method, on Dengue Fever Distribution in Jeddah City, Saudi Arabia

**DOI:** 10.3390/ijerph18126407

**Published:** 2021-06-13

**Authors:** Ibrahim Alkhaldy, Pauline Barnett

**Affiliations:** 1 Department of Administrative and Human Research, Umm Al-Qura University, Makkah 21955, Saudi Arabia; 2 School of Health Sciences, University of Canterbury, Christchurch 8140, New Zealand; pauline.barnett@canterbury.ac.nz

**Keywords:** dengue fever, socio-economic status, Delphi method, GIS, Jeddah city

## Abstract

Dengue fever, a mosquito-transmitted viral disease, is present in many neighborhoods in Jeddah City, Saudi Arabia. One factor likely to affect its distribution is the socio-economic status of local neighborhoods; however, the absence of socio-economic census data in Saudi Arabia has precluded detailed investigation. This study aims to develop a proxy measure of socio-economic status in Jeddah City in order to assess its relationship with the occurrence of dengue fever. The Delphi method was used to assess the socio-economic status (high, medium or low) of local neighborhoods in Jeddah City. A Geographic Information System (GIS) was applied to understand the distribution of dengue fever according to the socio-economic status of Jeddah City neighborhoods. Low-socio-economic status neighborhoods in south Jeddah City, with poor environmental conditions and high levels of poverty and population density, reported most cases of dengue fever. Nevertheless, dengue continues to increase in high socio-economic status neighborhoods in the northern part of the city, possibly due to ideal breeding conditions caused by the presence of standing water associated with high levels of construction. Moreover, the low-socioeconomic-status neighborhoods had the highest average number of cases, being 3.95 times that of high-status neighborhoods for the period 2006–2009. The Delphi approach can produce a useful and robust measure of socio-economic status for use in the analysis of patterns of dengue fever. Results suggest that there are nuances in the relationship between socio-economic status and dengue that indicate that higher status areas are also at risk. A useful additional tool for researchers in Saudi Arabia would be the development of census data or other systematic measures that allow socio-economic status to be included in spatial analyses of dengue fever and other diseases.

## 1. Introduction

Dengue fever is a viral disease transmitted from person to person via the *Aedes aegypti* and *Aedes albopictus* mosquitoes [1]. It can be caused by any one of four serotypes that can lead to severe influenza-like symptoms, often with debilitating and long-term consequences [2]. About 3.9 billion people, in 128 countries, are at risk of infection [3], with an estimated 390 million dengue infections occurring annually, of which 96 million are recognized clinically and include 20,000 deaths [4,5]. Global Burden of Disease 2013 revealed a 610% increase in incidence since 1990, the highest of any of the so-called ‘neglected tropical diseases’ (NTDs) [6]. Surges in cases internationally are considered to be due to the growth of urbanization, increased global travel and climate factors [7,8].

The dispersal of *Ae. aegypti* and the emergence of dengue fever have been associated with the development of rural water supply structures and improved transport systems [9]. Mosquitoes are most often seen in urban areas where there is poor water management, the presence of non-degradable tires and plastic containers (where standing water accumulates), as well as inaction of the public health community to eliminate mosquito breeding sites [10].

The eggs of *Ae. aegypti* are laid by adult females on wet surfaces, just above the water line [9]. In non-urban environments, the breeding sites are located in places that resemble tree-hole settings and tree stumps. In urban habitats, the mosquitoes lay eggs on small artificial containers, such as cans, buckets, flower pots and bottles in people’s yards and inside houses [11]. The eggs can survive without water for long periods of time, sometimes for more than a year, and this enables the species to survive during adverse climatic conditions [9].

Dengue is a serious public health problem associated with neighborhood level physical and social environmental conditions [12]. The desert environment of much of the Arab world would be expected to be unfavorable to dengue fever, with Saudi Arabia considered dengue free until the 1990s. In 1994, 289 cases were identified in Jeddah City, located on the more humid Red Sea coast [13,14], but with other parts of the country experiencing seasonal outbreaks [13,14,15,16,17,18]. The presence of dengue in Jeddah City is important; this city is the gateway for Hajj pilgrims to Mecca, creating population-level vulnerabilities. Recently, the importance of this has been observed internationally in the need to manage disease risks in large gatherings [19,20].

Dengue fever is associated with both general climatic conditions and a range of other factors [21], including levels of urbanization and socio-economic and built environments. While international research has associated socio-economic status with dengue fever [22], spatial analysis of local neighborhood characteristics and dengue fever offer additional insights into causes and opportunities for prevention [23]. Unfortunately, no socio-economic data are available for Jeddah City at the neighborhood level, and so undertaking any such analysis requires the development of proxy measures. This research aims to develop a proxy measure of socio-economic status in Jeddah City and apply this to understand its association with the occurrence of dengue fever.

## 2. Materials and Methods

### 2.1. Study Area and Data Sources

Jeddah City lies almost mid-way up the western coast of Saudi Arabia. It is bordered by the Red Sea on the west and the Al Sarawat Mountains in the east. There are no rivers or valleys in the area. Due to its location, Jeddah City is the second commercial center of the Middle East after Dubai, and it is also the fourth largest industrial city in Saudi Arabia after Riyadh, Jubail and Yanbu. This section describes the data sources and methods used to undertake a spatial analysis of the relationship between dengue fever and socio-economic status in Jeddah City for the period 2006–2009. Three data sources were used:

Population data. According to the 2010 national official census of Saudi Arabia, the population of Jeddah City is 3,457,794 [24]. The city is divided into 112 neighborhoods. In 2009, the largest neighborhood was Al Safa with a population of 224,398, whereas the smallest, Al Senaeya, had a population of 853 [25]. Official census data for Jeddah City neighborhoods are only available from the Central Department of Statistics and Information for 2004. The next census in 2010 provides total population data for Jeddah City, but no data at the neighborhood level [24]. The Jeddah Urban Observatory in Jeddah Municipality has estimated the population of selected Jeddah City neighborhoods for 2009 [25]. The census data for 2004 and the population estimates for 2009 were used to estimate neighborhood populations between those years. There were 56 neighborhoods for which population data could be established.

Dengue fever data. Data on dengue fever cases from 2006 to 2009 by the neighborhoods for Jeddah City were provided by the Health Ministry in Saudi Arabia. While dengue cases from 2006 to 2009 could be matched to all neighborhoods, as previously noted, there were only 56 neighborhoods for which census data were available. These neighborhoods contained 74.7% of all dengue fever cases in 2006, 65.8% in 2007, 75.8% in 2008 and 63.6% in 2009.

Socio-economic data. As previously noted, even population data for Jeddah City neighborhoods are incomplete, and no socio-economic information is available. The building quality information reported by Khormi and Kumar (2011), while useful, does not represent an assessment of socio-economic circumstances [26]. As an alternative, this study used a modified Delphi approach, a qualitative research method, to develop a socio-economic status measure to enable an analysis of the spatial relationship between dengue fever and socio-economic status in Jeddah City. Both the Delphi method and the spatial analysis are described below.

### 2.2. Research Methods

Delphi Method. The Delphi method uses a panel of experts whose opinions are elicited on a research question or area of study. The experts’ opinions can provide optimal clarification of the area of enquiry and achieve a high degree of reliability [27]. This method has been used widely to develop a consensus on various topics [28,29], including selecting healthcare quality indicators [30]. This is the first time, however, that the Delphi method has been used in the Middle East to develop an assessment of neighborhood socio-economic status. The Delphi method depends on a continuous cycle of questioning, collecting information, summarizing and then refining the experts’ opinions to provide a focus on the main concerns of the researcher [27].

To apply the Delphi method to this study, a checklist was sent by e-mail to 32 specialists who worked in Jeddah City and had professional knowledge of its neighborhoods. These specialists were contacted through their organizations, and of the 25 who replied, 11 were from a Government agency, 10 from academia and four were from a financial institution. In brief, the checklist invited them to describe the socioeconomic status of individual Jeddah City neighborhoods. They were provided with a list of neighborhoods, linked to a map to show the location for each neighborhood, and asked to assess the neighborhood as low, middle, or high status. No guidance or criteria for the selecting categories were given. This approach was chosen so that respondents had the freedom to consider all the factors they thought to be helpful in making an assessment. The participants completed the checklist and e-mailed it back.

Once the completed checklists were received, the 25 responses were tabulated according to the frequency of a high, middle, or low socioeconomic status rating for each neighborhood. These rankings were used to derive a score based on the percentage of ranks in each socioeconomic status category. The highest percentage of any socioeconomic status category was chosen as the socioeconomic status ranking of the neighborhood. These results were then sent to the respondents for comments, and most of them agreed with the results as presented. For those who disagreed, they believed that some of the middle socioeconomic status neighborhoods contained areas that more closely resembled low-status neighborhoods reflecting their close proximity to these areas. To demonstrate the socioeconomic status level of the neighborhoods, a field study was undertaken to document the quality of housing, buildings and social environments that characterizes socioeconomic classifications from 1 (high) to 3 (low).

Spatial analysis. The result from the Delphi method was used to map the socio-economic status for the neighborhoods in Jeddah City and clearly identify where they were located.

For each of the four years (2006, 2007, 2008 and 2009), dengue cases were allocated to groupings of low, middle and high socio-economic status neighborhoods in Jeddah City. An average for all four years was also calculated for each socio-economic neighborhood grouping. In addition to the total number of cases, rates per 10,000 population were calculated for each socio-economic neighborhood grouping for each year and as a four-year average. Means and standard deviations were calculated for both total cases and rates per 10,000 for each socio-economic grouping.

In addition, to understand the distribution of dengue fever in Jeddah City neighborhoods, from 2006 to 2009, the average of 2006–2009 and the number of dengue fever cases per 10,000 people for each year in Jeddah City neighborhoods were mapped. This distribution of dengue fever cases among various socioeconomic levels of Jeddah City neighborhoods will help to describe these relationships in each socioeconomic situation.

### 2.3. Results

**Figure 1:**
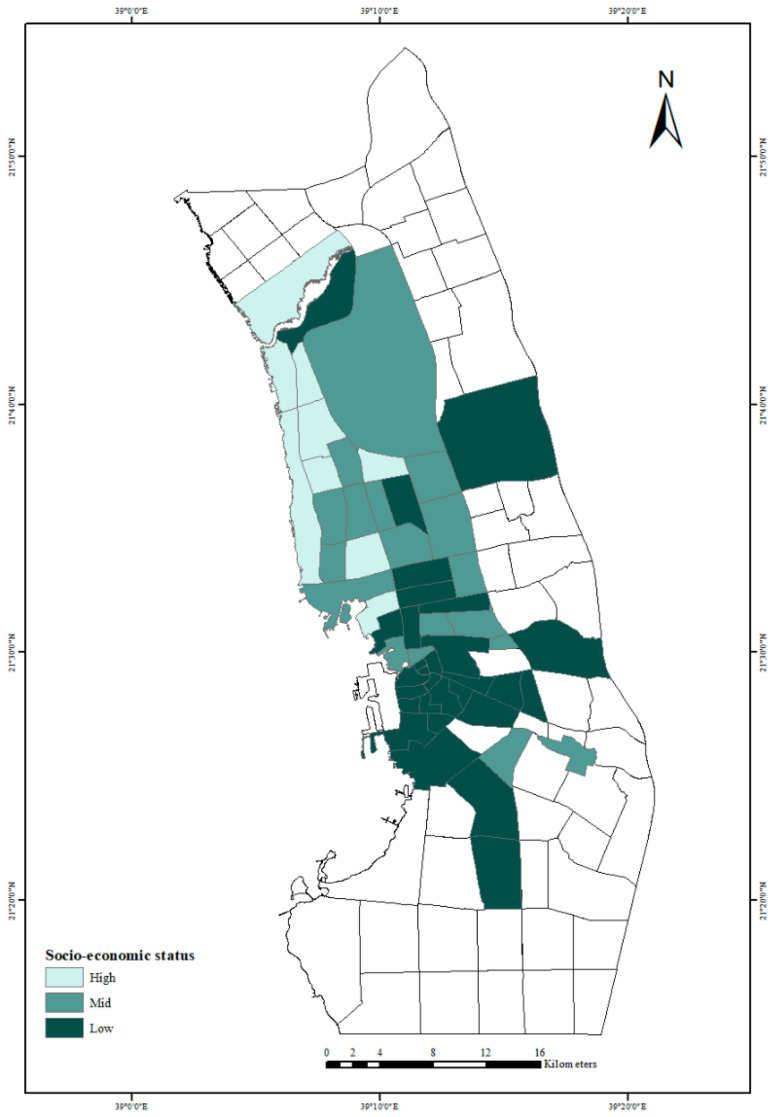
Socio-economic status of neighborhoods in Jeddah City.

Socio-economic measures. Figure 1 shows the socio-economic categorization of Jeddah City neighborhoods. Most of the neighborhoods in the south of Jeddah City are of low socio-economic status, while the high socio-economic status areas are located in the middle and west of the city near to the Red Sea. Middle socio-economic status neighborhoods are mainly in the north and are mostly new neighborhoods. Of the 56 neighborhoods, 9 are of high socio-economic status, 20 are of mid-status and 27 are classified in the low socioeconomic status category.

The field study found that the housing conditions in the high-socioeconomic-status neighborhoods (Level 1) are very good, typically comprising villas built for one or two families. There are also leisure areas located close by, including sport fields and recreational facilities. These neighborhoods typically have a low population density and streets that accommodate two or three car lanes. These neighborhoods have good-quality water and sewage networks and pipes. Most people living in these neighborhoods are Saudi.

The middle socioeconomic status neighborhoods (Level 2) are different, with housing comprising predominantly apartment buildings located near to market areas. Most of these neighborhoods are quite high density and predominantly Saudi, with more than 6–8 people (including one non-Saudi house-worker) living in a 4–5-bedroom apartment. There are difficulties parking in these neighborhoods as each family owns on average 2–3 cars. Due to the higher population density, water and electricity supply may be interrupted, especially in the summer season, due to the demand for air conditioning [31].

The poorest housing and living conditions in Jeddah City are in the low socio-economic status neighborhoods (Level 3). These neighborhoods tend to be old, with the residents predominantly being non-Saudi. Due to the low cost of housing, these are the only areas where poor non-Saudis can afford to live. The neighborhoods, located in central and southern Jeddah City, have high population densities, and at times, electricity or water is unavailable [31]. People, therefore, resort to saving water in tanks to use when needed. The drainage network is not adequate and only a small amount of rain is needed for surface water to appear.

Spatial analysis. The neighborhood variations in the number of dengue fever cases and the rate per 10,000 people for the 56 neighborhoods in Jeddah City grouped by neighborhood socioeconomic status is shown in Table 1. Low-socioeconomic-status neighborhoods had the highest average number of cases, being 3.95 times that of high-status neighborhoods for the period 2006–2009. Social differences in the number of cases, as measured by the ratio of the average number of cases in high compared to low-socioeconomic-status neighborhoods, were greatest in 2008, but also high in 2009. While low-socioeconomic-status neighborhoods had the highest average number of cases, the high standard deviations, nevertheless, indicate that substantial variations existed even among low-status areas. As expected, the standard deviations for the number of cases are higher than those for case rates. With the exception of 2006, internal neighborhood differences, regardless of socioeconomic status, in the number of cases and case rates tended to increase over time.

Neighborhood variations in rates of dengue fever also show some social patterning but are much less marked when compared to variations in the average number of cases. When actual rates are compared between low- and high-status neighborhoods, a social gap in case rates also occurred in high-status neighborhoods. The reason for the high number and rate of dengue fever cases in the low-socioeconomic-status neighborhoods reflects a number of factors, including poorer environmental conditions, large numbers of migrant people, high population densities and poor lifestyles. These factors are also conducive to the rapid spread of dengue fever cases in Jeddah City neighborhoods.

**Figure 2:**
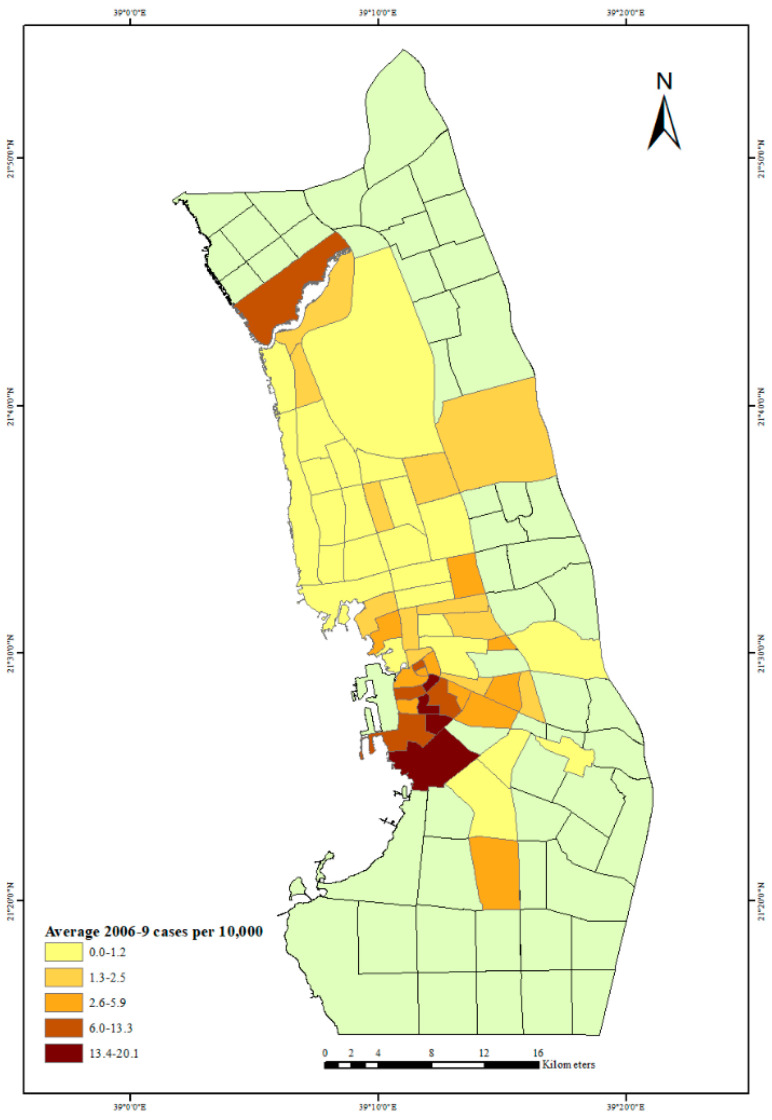
Average dengue fever cases per 10,000 people in Jeddah City, 2006–2009.

As shown in Figure 2, the areas with the highest average number of dengue fever cases per 10,000 people from 2006 to 2009 are located in the city center and southern part of Jeddah City, which are the less affluent parts of the city. There is only one neighborhood with a high rate of dengue fever cases that is located outside of central and southern Jeddah City; this is Obhur Al Shamaliah, a high-status neighborhood with a small population. As it is a new neighborhood, there is a high rate of construction, providing many opportunities for dengue to occur.

**Figure 3:**
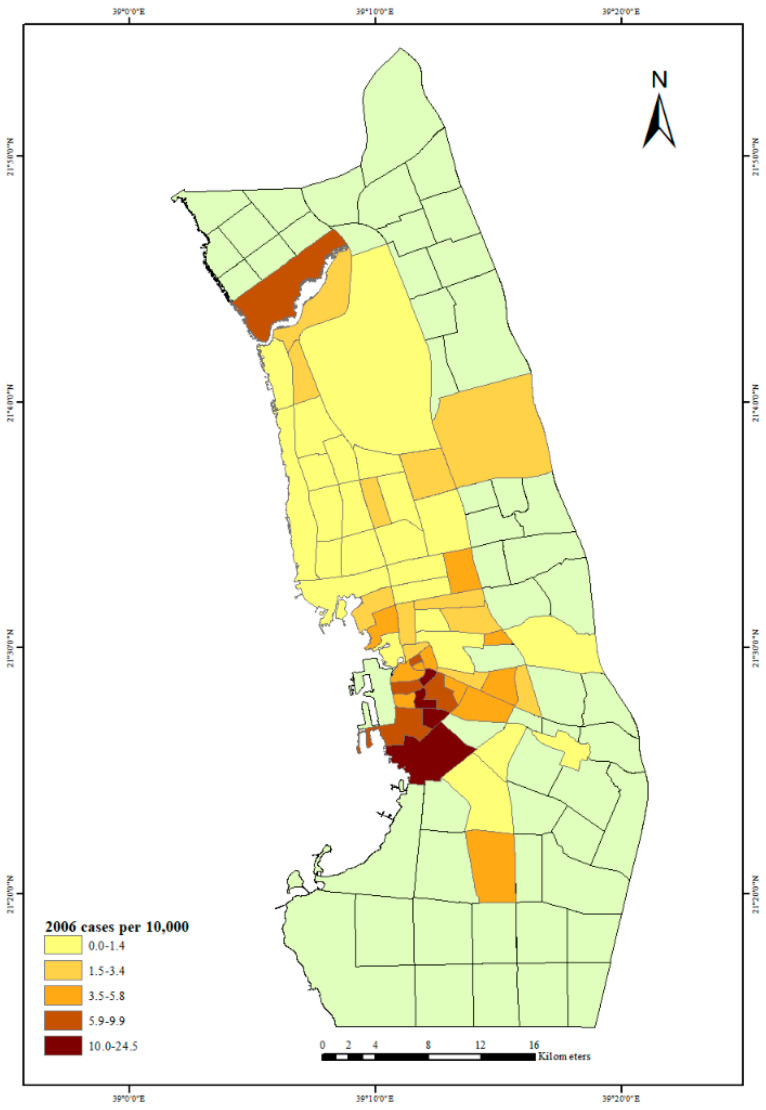
Dengue fever cases per 10,000 people in Jeddah City, 2006.

**Figure 4:**
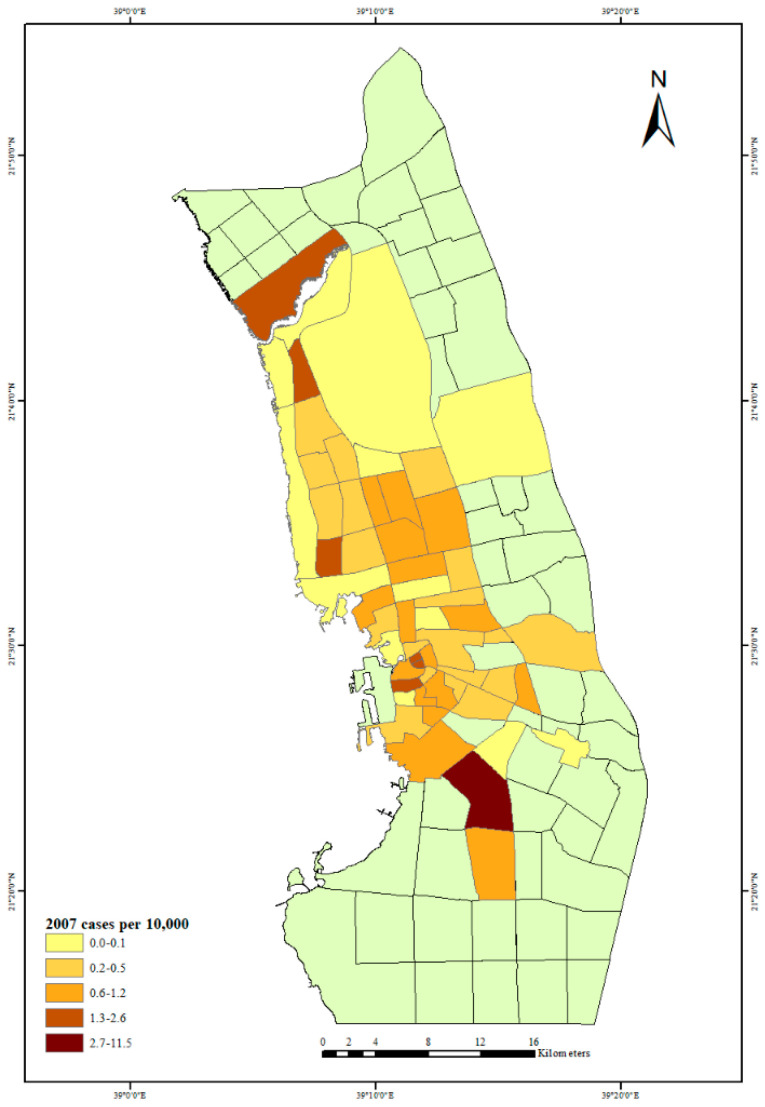
Dengue fever cases per 10,000 people in Jeddah City, 2007.

**Figure 5:**
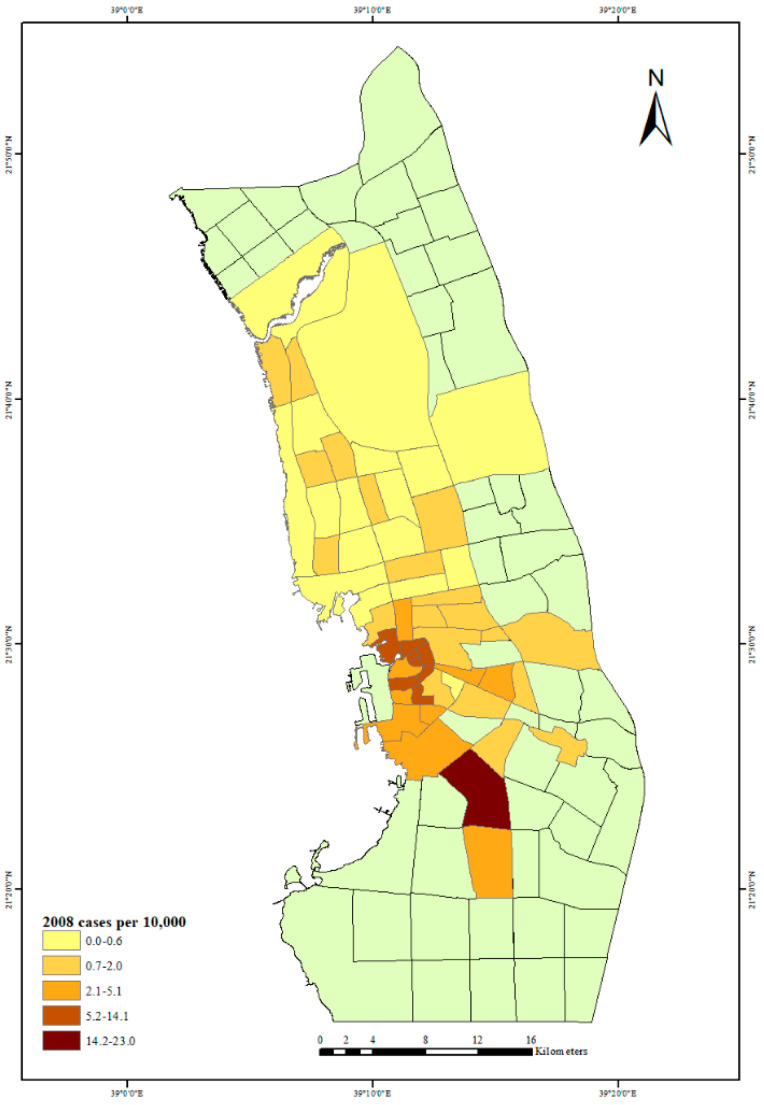
Dengue fever cases per 10,000 people in Jeddah City, 2008.

**Figure 6:**
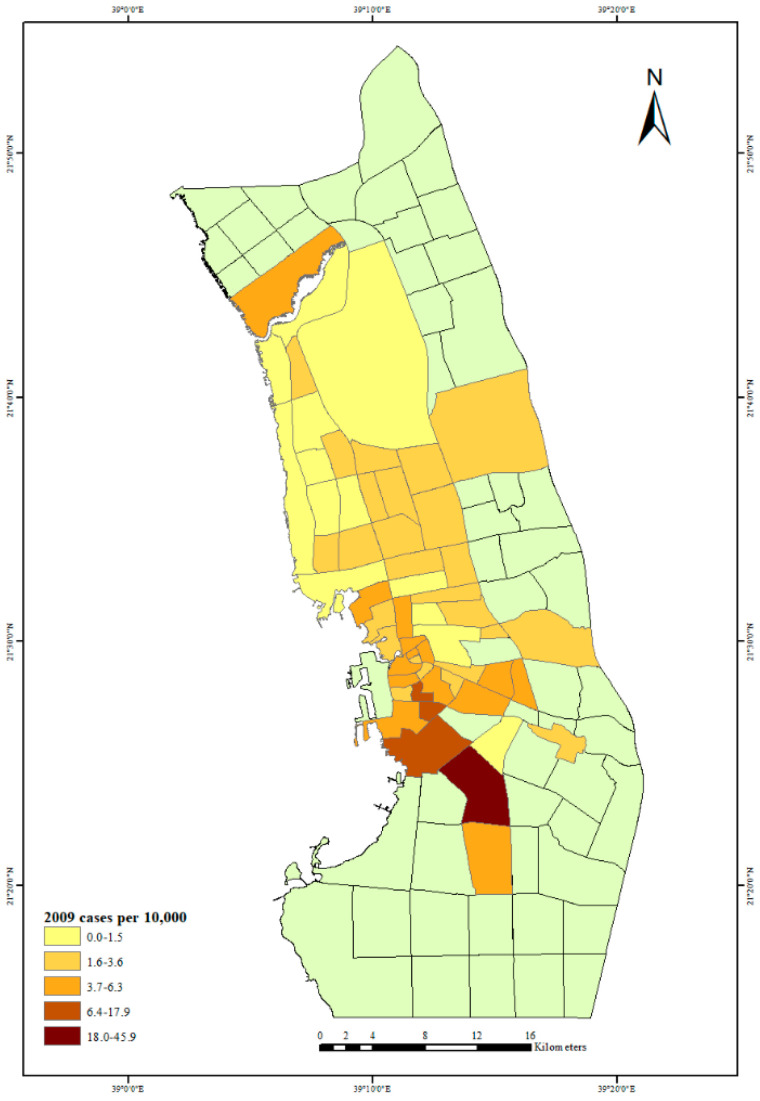
Dengue fever cases per 10,000 people in Jeddah City, 2009.

The first outbreak of dengue fever in Jeddah City in 2006 is depicted in Figure 3. The neighborhoods with the largest number of dengue fever cases per 10,000 people can be seen in the city center and north of the city. The highest rate of dengue fever cases was in the center of the city, but in 2007, there was also a high rate of dengue fever cases north and south of the city center; see Figure 4. The years 2006 and 2007 had almost the same distribution of dengue fever, but in 2008, there was a high rate of dengue fever cases in the city center and in one neighborhood to the south of the city center (Figure 5). By 2009 there was also evidence of dengue spreading into neighborhoods north of the city center (Figure 6).

## 3. Results and Discussion

The main objective of this study was to identify the relationship between the patterns of dengue occurrences and the socioeconomic status of Jeddah City neighborhoods. Since there has been no official socioeconomic status measurement of the census for Jeddah City, or any other Saudi city, the Delphi method was used to develop an alternative indicator of socioeconomic status for Jeddah City neighborhoods, which was then used in this research. This innovative approach was supported by on-site visits of Jeddah City neighborhoods. There were some limitations to the Delphi procedure. For logistical reasons, the checklist questionnaire was kept simple; the number of respondents was relatively low and the feedback process fairly rudimentary. A photographic record of typical neighborhoods was only partly successful as there were safety issues for the researcher in poorer areas. Despite these limitations, the Delphi method worked well, could easily be improved and provided more context than, for example, remote sensing methods. Furthermore, the Delphi method could also be useful in developing a consensus on strategies for dengue control.

The spatial analysis of dengue fever cases in Jeddah City neighborhoods found, as expected, that the greatest number of dengue cases in the low socioeconomic status areas occurred in the center and southern part of Jeddah City, known as “old” Jeddah City. Old Jeddah City has a large population, and there are neighborhoods with a significant percentage of foreign migrants living in poor conditions at high population densities, and it has developed into an ideal place for immigrants to live as there is cheaper housing. These patterns are similar to those found in another study of dengue in Jeddah City, which also showed dengue cases to be concentrated in the city center and southern parts of the city [32].

The high-socioeconomic-status neighborhoods which have a lower rate of dengue fever cases are located in the north of Jeddah City, with their lower rates attributed to better environmental conditions and low population densities in newer neighborhoods. Nevertheless, even there, more cases appear every year. Between 2006 and 2009, the distribution of dengue fever cases in Jeddah City neighborhoods became more widespread, and by 2009, dengue fever could be found almost everywhere in Jeddah City. It has been suggested that the high levels of construction and the use of concrete requiring large quantities of water that was often found to be standing in construction sites have created a conducive environment for mosquitos. Jeddah City authorities hold unpublished field study information, sighted by IA, on mosquito presence in various settings. These conditions may have contributed to the increase in cases in high socioeconomic status areas.

We noted above the growing interest in geospatial analysis as a tool for the epidemiology of dengue fever. An important component of this approach is understanding the impact of scale and how variables appearing important at one scale may be less so at another [33]. Despite this interest, Guo et al. (2017) identified only around 20 articles between 1990 and 2015 that addressed the spatial components of dengue fever outbreaks [34]. Marti et al. (2020) reviewed the literature and created an inventory of the most relevant landscape factors for dengue transfer in urban areas [35]. They identified 78 articles that met quality and content criteria and then structured their review according to geographic context, epidemiological descriptors, landscape factors, and evidence of a relationship between urban determinants and dengue cases [35]. This study emphasized the need to consider multiple variables, given the complexity of the ‘pathogenic landscape’ and to find ways of using remote sensing to describe intricate urban environments, minimizing the need for costly and time-consuming surveys [35]. They concluded that an integrated approach, combining remote sensing, GIS and field surveys is desirable, with health data and entomological observation likely to remain limiting factors [35].

## 4. Conclusions

Perhaps the highest priority in Saudi Arabia in terms of future research is the development of more comprehensive neighborhood demographic and socioeconomic indicators that are known risk factors for dengue. Ideally, as social and demographic conditions in Jeddah City are in a state of constant change, a temporal analysis of such indicators is also important [36,37]. Research studies, including GIS and remote sensing, all have a part to play, but perhaps improvements in the scope and execution of the census could provide more accurate and recent social and environmental data that could help in analyzing outbreaks and targeting dengue control policies. Studies into the presence of mosquitoes in water sources, in addition to research into the responses of different socio-economic groups in the community to the threat of dengue fever, will also provide important insights to improve the management of dengue fever risks.

## Figures and Tables

**Table 1:** Average number of dengue fever cases and case rate per 10,000 people by neighborhood socioeconomic status in Jeddah City, 2006–2009.

Year	Mean			Standard Deviation		
	High SES	Mid SES	Low SES	High SES	Mid SES	Low SES
Cases, 2006	11.2	7.0	27.2	16.8	7.3	30.8
Cases, 2007	1.1	2.00	4.0	0.8	3.1	4.5
Cases, 2008	1.6	5.8	17.8	1.4	7.3	16.4
Cases, 2009	5.4	11.7	27.3	5.9	15.1	23.8
Average Cases, 2006–2009	4.8	6.6	19.1	5.2	6.9	15.3
Rate cases, 2006	6.2	2.4	6.5	11.5	3.2	7.5
Rate cases, 2007	0.7	0.3	1.2	0.9	0.4	2.4
Rate cases, 2008	0.5	1.9	4.7	0.4	3.6	5.3
Rate cases, 2009	6.0	3.1	6.7	8.3	7.1	9.2
Average rate cases, 2006–2009	3.4	1.9	4.8	4.7	3.9	4.5

## Data Availability

The data presented in this study are available on request from the corresponding author.
